# RETRACTION: Amelioration of Ovalbumin‐Induced Allergic Asthma by *Juglans regia* via Downregulation of Inflammatory Cytokines and Upregulation of Aquaporin‐1 and Aquaporin‐5 in Mice

**DOI:** 10.1155/jotm/9801423

**Published:** 2026-05-11

**Authors:** Journal of Tropical Medicine

RETRACTION: M. Sharif, I. Anjum, A. Shabbir, et al., “Amelioration of Ovalbumin‐Induced Allergic Asthma by *Juglans regia* via Downregulation of Inflammatory Cytokines and Upregulation of Aquaporin‐1 and Aquaporin‐5 in Mice,” *Journal of Tropical Medicine,* no. 2022 (2022), https://doi.org/10.1155/2022/6530095.

The above article, published online on 30 March 2022 in Wiley Online Library (https://wileyonlinelibrary.com), has been retracted following the agreement of the journal’s Academic Editor, Rajib Chowdhury; and John Wiley & Sons Ltd.

The retraction has been agreed following an investigation into concerns initially raised by *Archasia belfragei* on PubPeer [1]. The investigation identified issues related to Figure 6, in which panels B and F in Figure 6a appear identical to panels B and F in Figure 3 of [2], where they represent a different experimental outcome. Further, panel F in Figure 6b shares similarity with Figure 4f of [2].

Subsequent investigation has also identified potential concerns in Figure 3, where unexpectedly similar features are seen in the Western Blots of IL‐13 and AQP‐5.

The authors responded to these concerns and provided raw data; however, concerns regarding the reliability of the information presented in the manuscript remain, and it is therefore retracted.

The authors disagree with this retraction.
